# The unseen patient: competing priorities between patients and providers when cannabis is used in pregnancy, a qualitative study

**DOI:** 10.3389/fgwh.2024.1355375

**Published:** 2024-04-18

**Authors:** Erin E. Gould, Siddhi S. Ganesh, Ryan Mikeala Nguyen, Carrie V. Breton, Theresa M. Bastain, Genevieve F. Dunton, Rachel Carmen Ceasar

**Affiliations:** Department of Population and Public Health Sciences, Keck School of Medicine, University of Southern California, Los Angeles, CA, United States

**Keywords:** pregnancy, prenatal cannabis use, healthcare provider knowledge, maternal health, substance use

## Abstract

**Objectives:**

This study aimed to explore competing priorities when cannabis is used during pregnancy from the perspective of providers and Black and Latina people. Maternal cannabis use is increasingly common, but patients and providers alike struggle to navigate it.

**Methods:**

This pilot used qualitative, constructivist ground theory methods to conduct semi-structured, remote interviews between 16 November 2021, and 7 February 2022 with 7 Black and Latina people who used cannabis during pregnancy, and 10 providers between 15 March 2022, and 6 April 2022, all of who were in Southern California, U.S.

**Results:**

We identified three main findings: (1) Providers reported barriers to caregiving and relationship building with patients due to maternal cannabis use stigma, (2) Providers prioritized the fetus despite patients’ current health system challenges that drove cannabis use, and (3) Both patients and providers engaged in personal research beyond the healthcare system to better understand maternal cannabis use.

**Discussion:**

Our findings indicate that challenges exist between people who use cannabis during pregnancy and providers. Both groups need accurate, sociocultural sensitive information about maternal cannabis use via a harm reduction lens.

## Introduction

Cannabis is the most common substance used in pregnancy (1). As early as the 7th century BCE, cannabis has been used as a medicinal treatment for ailments specific to reproductive and gynecological events (2), and people today continue to use it for pregnancy-related symptoms such as nausea, pain, and mood changes (3–6). Pregnant and postpartum individuals are increasingly using cannabis, especially in states where it is legalized (7–9). In recent years, estimated rates of use are up to 8% in this group and may even be higher due to lack of self-report data caused by fear of stigma and legal consequences (10–12, 5, 6, 13, 14).

The American College of Obstetricians and Gynecologists (ACOG) and the American Academy of Pediatrics (AAP) (15) advise against cannabis use during pregnancy because there is convincing evidence from observational studies that people who use cannabis during pregnancy are more likely to give birth to babies that are small for their gestational age and have a low birth weight (16). Specific cannabinoids present in cannabis can be effective for multiple sclerosis and chronic pain treatment (16) but it is likely that *in utero* exposure to cannabis may produce negative health effects (17, 18). However, there are limitations to the conclusiveness of the current body of evidence (19). Limitations of current evidence include sample size and generalizability, self-reported data, lack of data on timing and dosing of cannabis ingestion, as well as a lack of focus on social environmental contexts influencing use of cannabis during pregnancy (20, 21). The extant data has identified that more research is needed on maternal cannabis use to optimize public health promotion and intervention development (22–24).

Pregnant individuals who are considering using cannabis report perceptions of insufficient data as well as not seeing providers as sources of information or care on this topic (24, 25). On the other hand, providers often describe a lack of confidence in their own knowledge of cannabis and the available medical protocol regarding maternal cannabis use, leading to issues with screening and counselling (5, 26–29). ACOG and other groups recommend universal screening practices for substance use in pregnancy, but screening is often non-standardized and is fraught with racial disparities (30). Patients of color are also the most likely to face punitive consequences for disclosures of maternal cannabis use such as Child Protective Services (CPS) involvement or stigmatized care, prompting criticism of screening practices (31, 32).

The paucity of reliable, high-quality data on the long-term effects of maternal cannabis use coupled with conflicting stances in healthcare settings can lead to potential confusion among both patients and practitioners (20, 25, 29). While more data is emerging, little is understood about how individuals navigate care settings while using cannabis during pregnancy or discussing maternal cannabis usage with their providers. Patients and providers alike often do not know what information to consult, apply, or trust when making an informed decision about cannabis use during pregnancy. This study aimed to explore competing priorities when cannabis is used during pregnancy from the perspective of providers and Black and Latina people who use or used cannabis during pregnancy. We explore the potential for ensuing inequities, such as selective substance use screening practices, that lead to disparities in care among Black and Latina pregnant people who use cannabis.

## Methods

### Study design—constructivist grounded theory

We conducted qualitative interviews with maternal healthcare providers as well as people who use cannabis during pregnancy, an approach that has been documented in our other studies (29, 33). The research team consisted of advanced public health students (RMN, SSG), research staff (EEG), faculty co-investigators (GFD, CVB, TMB), and a faculty member (RCC) acting as the Principal Investigator of the study. We reviewed COREQ (consolidated criteria for reporting qualitative research), a validated 32-item checklist for qualitative reporting throughout the study to document important aspects of our research team, methodology, findings, and analysis (Supplementary Table S7: COREQ checklist) (34, 35).

We conducted a phenomenological study using constructivist grounded theory that aimed to understand the perspectives of two groups who have experienced a shared phenomenon, respectively: maternal health providers who care for patients who use cannabis during pregnancy and non-Hispanic Black and Latina people who used cannabis during pregnancy (36, 37).

### Selection of participants—sampling and recruitment strategies

We used theoretical sampling based on grounded theory methodology (38, 39). This study is part of a larger project focused on patient and provider experiences regarding maternal cannabis use (40). We recruited patients from the Maternal and Developmental Risks from Environmental and Social Stressors (MADRES) cohort study who enrolled pregnant persons from three locations that predominantly serve patients with Medi-Cal, a California Medicaid health program that directly pays for medical services for people with limited income through federal and state taxes (40, 41). The study coordinator contacted patients from the larger MADRES cohort who were identified as using cannabis during pregnancy through medical record abstraction; had given birth in the last 0–2 years; were 21 years or older; and fluent in English or Spanish (Table 1). We recruited maternal health providers (e.g., physicians, doulas, midwives) via selective sampling using a convenience sample and snowball approach to reach practitioners in safety net health settings in Southern California who had experience caring for people who use cannabis during pregnancy within the past year (Table 2). This type of non-random sampling was based on team discussions about selecting participants that would provide insightful information regarding the research question (42, 43). Potential participants completed a HIPAA-compliant REDCap survey to confirm eligibility, sign an e-consent form, and schedule an interview.

**Table 1:** Study participant characteristics (pregnant patients, *N* = 7).

	Mean (SD)/Frequency (%)
Participant characteristics	
Age	27.24 (3.39)
Nativity	
* *Non-hispanic	3 (42.86%)
* *US-born hispanic	3 (42.86%)
* *Foreign-born hispanic	1 (14.29%)
Education	
* *Completed grade 12 (high school)	2 (28.57%)
* *Some college or technical school	3 (42.86%)
* *Completed 4 years of college	2 (28.57%)
Income	
* *Don't know	2 (28.57%)
* *Less than $15,000	1 (14.29%)
* *$15,000–$29,999	2 (28.57%)
* *$30,000–$49,999	2 (28.57%)
Preferred language	
* *English	7 (100%)
Hispanic ethnicity	
* *No	3 (42.86%)
* *Yes	4 (57.14%)
NIH race categories/ethnicity	
* *Black, non-hispanic	3 (42.86%)
* *Hispanic	4 (57.14%)

**Table 2:** Study participant characteristics (maternal healthcare providers, *N* = 10).

	*n*	(*n* %)
Maternal health provider, cares for BIPOC (Black, Indigenous, and People of Color) pregnant people	10	100%
Cares for		
People who use cannabis during pregnancy	10	100%
People who use alcohol or other substances during pregnancy	9	90%
People who use cannabis after pregnancy	10	100%
Maternal health role		
Doula/lactation consultant	1	10%
Certified nurse midwife	1	10%
Physician	8	80%
Specialty		
OB/GYN	9	90%
Labor and delivery	2	20%
Postpartum	2	20%
Community health	1	10%
Midwifery	1	10%
Doula	1	10%
Lactation consultant	1	10%
Maternal health provider's racial identity		
Black or African American	2	20%
White	7	70%
Mixed race	1	10%
Maternal health provider's pronouns		
He/him	2	20%
She/her	7	70%
Declined to disclose	1	10%
Maternal health provider's age		
Under 30	2	20%
30–39	3	30%
40–49	3	30%
50–59	2	20%

### Data collection—semi-structured interviews

The semi-structured interview guides drew upon existing qualitative and quantitative literature (Supplementary Table S1: Interview guide 1, Supplementary Table S2: Interview guide 2) (27, 44–46). We piloted and adapted the questions within the research team. We revised the interview guides as the interviews progressed to refine questions and pursue areas identified as theoretically relevant. This was part of an iterative process aimed at generating richer responses from participants (39, 47).

The 60-min interviews occurred remotely via HIPAA-compliant Zoom™ video calls from 16 November 2021 to 7 February 2022 (patients, *n* = 7) or from 15 March 2022 to 6 April 2022 (providers, *n* = 10). Each interview was conducted by 1–3 research team members, with one individual leading the discussion and the others co-leading and taking analytical notes to inform analysis. We followed up on questions with open-ended inquiries about topics introduced by the participants. This non-directive, open-ended approach of qualitative interviewing encouraged participants to elaborate beyond the original scope of the interview guide and allowed for unanticipated perspectives (48). Further, the interview guide was iteratively revised based on topics introduced by participants to generate better insights in subsequent interviews (39). Throughout data collection, the theoretical sampling strategy guided responsiveness to emerging theory to identify important concepts and guide decisions about when data collection was complete (38, 39).

We sent audio recordings of interviews to an external transcriptionist who de-identified transcripts and then uploaded the files to a HIPAA-compliant One Drive for team analysis. The team made summaries of emerging ideas in transcripts after each interview was completed as part of the initial analysis. As data collection continued, emerging ideas aligned with previously observed phenomena, meaning that theoretical saturation was achieved (38, 47).

### Data analysis—grounded theory

We used grounded theory methodology to generate a conceptual framework (theory or explanation) by analyzing and comparing data on experiences and discussion around an area of interest (maternal cannabis use) across the data (38). This method is best used to learn about social processes when little is known about a phenomenon (39). Once the final interview was completed, the team reviewed and categorized the summarized emerging subject areas in each transcript to develop codebooks for patient and provider datasets. The codebooks consisted of thematic categories with definitions and examples discussed and revised as a team. We then uploaded the transcripts into ATLAS.ti™ data software program, Mac Version 22.1.0, and inputted the codebooks, which were tested on one transcript from each dataset as a team and revised as needed, resulting in 16 thematic codes for each patient and provider dataset (Supplementary Table S3: Codebook 1, Supplementary Table S4: Codebook 2) for analysis (49). Then, two team members independently analyzed each transcript and assessed consistent code application to ensure intercoder reliability. Final memos were made to capture themes resulting from code overlap and to facilitate deeper discussions of the data. Final insights from this process developed into the three results as they pertain to codes and final memos related to patient-provider relationships, priorities, and access to information.

This was a phenomenological study which used constructivist grounded theory to generate results. Constructivist grounded theory is a methodology where themes are inductively generated from the data and we develop specific theories and constructs as they relate to each other, co-occur, and build upon each other to add contextual richness to our data. As such, this methodology does not necessitate results with equal numbers of each group of participants, comparison within groups, or quotes from all participants and is instead focused on demonstrating results that best represent our themes. We did not include quotes from certain providers (i.e., doulas and midwives) because the quotes chosen were the best representation of the themes presented in results. While using theoretical sampling, we reached theoretical saturation for both patient and provider cohorts indicating that our results rigorously reflect and evaluate both perspectives (38, 47).

## Results

### Result 1: providers reported barriers to caregiving and relationship building with patients due to stigma around maternal cannabis use

Most providers described the prevalence of judgement towards cannabis in medicine today. One provider recounted their appraisal of patient disclosures, noting that most patients won't disclose cannabis use to their provider in fear of judgement:

“[T]here's still a decent amount of judgment… medicine is historically and traditionally patriarchal and … dismissive … the remnants of that circulate still … [patients are] not going to tell their doctor they’re using cannabis … because we can occasionally be judgmental, or we have a history of being judgmental.” (Dakota, Physician—OB/GYN)

Providers further explained this judgmental history via discussion of enacted screening practices that cause patients who disclose cannabis use to face negative repercussions. They explained that provider views of cannabis use leading to other substances (i.e., the gateway theory) meant patients attempting to communicate openly with providers are not only judged but subjected to punitive treatment as well:

“[C]annabis is seen … as a gateway drug in our particular specialty [inpatient maternity nursing] … we see that as a risky behavior … it does feel a little punitive … because … the person who's not saying anything [disclosing cannabis use] gets a different treatment [lack of drug screening] … than somebody who's trying to be honest about what is actually occurring.” (Cameron, Women's Health Nurse)

One provider recounted socioeconomic differences in screening and care: at different care sites, lower-income patients were monitored for substances without any evidence of use, but higher-income ones were spared this surveillance. They detailed provider assumptions about patients' behavior and how that impacted which ones received more oversight:

“[I]n the private practice, it's definitely a bias among the other providers that women with money don't have these issues … when I was at the low-income clinic … they would just run a drug panel on everybody in every pregnancy. And people would get dinged for using weed. We wouldn't call social services or anything because it wasn't a reportable drug use, but then they would get tested in labor at the hospital … in private practice, the doctors just figure that ‘Oh, women with money know better [than to use cannabis in pregnancy], so they don't do it.’” (Avery, Nurse Midwife)

They detailed the judgement and lack of understanding exhibited by many providers towards pregnant patients who are using cannabis. They called for divesting from this thinking via examination of patient motivations for using cannabis:

“[A]mongst the nurses, they … thought that the patient was just a shitty person for exposing their baby to drugs, and not looking at it as this whole entire problem … ‘Well it's really not that bad, but you should know better. It's not a huge addiction, like methamphetamine or heroin is. Why can't you just stop?’… [providers] looked down on the women for using it, not asking why they were using it. ‘Cause that's how I use it. If you're using cannabis, why? Is it because you have anxiety, is it because you have stress, is it because you have nausea?” (Avery, Nurse Midwife)

Another participant described the patient stereotypes many providers hold and emphasized the importance of exposure to patients who use cannabis to combat them. Without the opportunity to directly challenge preconceived notions regarding patients' views on pregnancy and their baby, they anticipated that the judgement towards cannabis use in pregnancy would continue:

“[I]f you practice in a population where you're not exposed to patients who are using cannabis, it can be a lot easier to maintain stereotypes about what kind of patient uses cannabis. And that only a patient who doesn't care about themselves, or doesn't care about their pregnancy, would use drugs during pregnancy.” (Riley, Physician—OB/GYN)

### Result 2: providers felt the need to prioritize the fetus and potential risks to it despite patients' current challenges within the health system that drove cannabis use

Patients and providers described a dissonance between their priorities as well as perceptions of risks and benefits of cannabis use. While patients wanted inclusion of their reasons for turning to cannabis in care conversations, providers expressed interest in following science that tends to focus on fetal outcomes rather than maternal ones. One provider detailed concern about the long-term impacts of prenatal cannabis use on fetal well-being and thus saw their role as using patient education to protect only the baby:

“[W]e have our unseen patient, which is our baby, that we're responsible for. The mom can be responsible for herself, but we [as inpatient maternity nurses] are responsible for that baby … It's not really for me to judge. It's really for me to educate [the mother]. And I follow the science. The science tells us that these babies can have problems [if they are exposed to cannabis prenatally].” (Cameron, Women's Health Nurse)

Yet, from the patient perspective, they felt “misjudged” by providers who prioritized potential risk of cannabis exposure to their fetus over the challenges they currently faced during pregnancy. One individual, who was using cannabis to deal with symptoms such as nausea and lack of appetite, shared difficulties with managing not only their pregnancy but also the perceptions of their providers as they explored the nuance of caring for self and baby in a health system with limited resources:

“They did give me the nausea pills, but they would make me throw up. So I didn't take those … they said they didn't have anything other than the pills … [People who use cannabis during pregnancy] get misjudged because they are … carrying another life. But you have to realize [what happens] … if they can't give to that life … [because] not all of the things [to manage pregnancy symptoms] that the doctor's office gives you works.” (Valeria, Patient)

Another participant expanded on what was lacking in their communication with providers as they trusted their own reasons for cannabis use. While their conversations were oriented towards the baby, this mother expressed a need to shift some focus to her own care and how to navigate that via harm reduction-based symptom management strategies:

“[Providers should] listen to their patients about why they use it while they're pregnant. I know they're concerned about the baby … but if it helps them in the first weeks of their pregnancy, as to morning sickness or they can't sleep … they should be allowed to use it at a certain point … give them a time limit or give them a deadline. Okay, when your morning sickness stops, don't use it.” (Emma, Patient)

Another provider described how mothers face punitive measures because of their cannabis use in pregnancy rather than being offered support. As providers decide from an individual standpoint to conduct precautionary screening and reporting of patients for using cannabis in pregnancy, they tie care to agencies that can potentially perpetuate harm. As a result, the relationship between patient and provider can be damaged:

“[Providers] will order drug screens, or will call DCFS, Department of Child and Family Services, if they're worried about somebody … cannabis use alone shouldn't trigger that. But it is kind of provider-dependent, whether they decide to order those tests or referrals. You're in this vulnerable situation … as a pregnant woman, where you're coming to a healthcare center, you're looking for care, and then some of the care, in this case, ordering additional drug screens, or calling in Child and Family Services (CPS) [due to cannabis use], isn't really aimed at helping the mom. It's potentially used against the mom, in terms of losing custody of the child. [I]t's … a betrayal of the provider/patient relationship.” (Riley, Physician—OB/GYN)

One provider similarly emphasized the support needed by patients and their role in this. Knowing why patients are turning to cannabis and counseling or providing resources specific to this shifts the focus to encompass both maternal and fetal needs:

“I think that recommendations … [are] less inclusive of the range of patient experiences. I find most recommendations … advise us to counsel patients to … stop all substances immediately. And there's not a lot of room for us to support patients in where they are in their life, and what that would mean to them if they stopped substance use, and how we could support them … I feel like definitely knowing and caring about patients who have some form of substance use really makes the difference in terms of how you look at it.” (Riley, Physician—OB/GYN)

### Result 3: both patients and providers felt the need to seek out information to better understand maternal cannabis use

Both patients and providers detailed seeking out information about cannabis use during pregnancy on their own. Patients described feeling compelled to do their own research amidst acknowledgment that healthcare providers were not open to their cannabis use. Reliance on peer knowledge and social media were commonly described among participants seeking information:

“My home girls told me, ‘Hey, I used it. Don't trip, it's fine. I know the doctor will try to scare you, but my mom's done it.’ … It's mostly just [learning from] word of mouth from friends. I know a couple [of canna] doulas on IG. She shares a lot of info.” (Mia, Patient)

Another participant recounted turning to their social networks to discuss maternal cannabis use and how these conversations were centered around the mother's experience:

“[A] few of my friends have had kids, and they smoked throughout their pregnancy … they were like, ‘Yeah, I think everything in moderation, do it because of your pain, not to get high.’” (Ava, Patient)

Providers acknowledged seeking out information on maternal cannabis use because they felt there was limited data on the subject but did not trust the knowledge patients shared about cannabis. One healthcare provider noted the challenge of navigating conflicting information when discussing patients doing their own research on maternal cannabis use:

“I've definitely learned things from patients in other areas, but with cannabis use, I would say probably not as much, just because there's not a ton of data out there that's on strictly cannabis use … somebody will quote a study … so I've gone and searched for that. But then at the same time, there's another study that says [the opposite] … point me into directions and I'll look it up, but it hasn't really changed anything.” (Avery, Nurse Midwife)

Another provider acknowledged that healthcare settings could take advantage of patients' drive to educate themselves and their social circles via peer-to-peer education programs, harnessing the positives of patients' desires and improving collective well-being:

“[T]hat'd be a big opportunity for our hospital as a whole to better connect with our community, is to empower them more to be leaders in the community and relay information that we give them to their peers and help them feel empowered that by improving their own health, they're also improving the health of their community as a whole.” (Taylor, Physician—OB/GYN)

## Discussion

Our findings showcase consequences related to cannabis use disclosure perpetuated by providers such as institutionalized stigma and structural violence (50), dissociation between prioritizing baby vs. pregnant patient when cannabis use is disclosed, and patient and provider perceptions of lacking information.

Structural violence, a conceptualization of social injustice, refers to the position of potential harm that individuals are put in due to inequities within large-scale forces; Social structures within economic, cultural, medical, and legal domains are embedded with inequities (i.e., race or class-based discrimination) which prevent the fulfillment of optimal health and well-being (50–54). This is a structural issue as these experiences are situated within social structures and it is violent because it causes harm. While healthcare providers often lack training and education on these structural forces, there is a need for providers to have a broader understanding of how health behaviors and outcomes are situated within the social context of the patient's lived experience (19, 45, 54). Our findings reinforce this need as providers in our sample lacked awareness of structural consequences that pregnant patients who use cannabis may face. Our data demonstrated that severe medicolegal consequences, ranging from drug screenings to CPS involvement, awaited patients who disclosed their cannabis use to their providers. Open and honest communication within the provider-patient relationship was disincentivized, whereas defensive communication was protective for the patient. These findings call attention to institutionalized stigma in medicine by subjecting patients seeking care at low-income clinics to increased surveillance via procedures such as drug screenings (55–58). This data demonstrates manifestations of structural violence as these structural inequities can result in relational tension between patients and providers as well as drastic consequences for patients such as family separation (50).

In our study, providers described prioritizing empirical data over patient lived experience when it comes to maternal cannabis use. Our findings indicate that providers rely on maternal cannabis use data which focuses largely or exclusively on fetal outcomes. Participants reported how patient-centered care practices including shared decision-making by factoring the needs of pregnant people and their reasons cannabis use were absent. We observed a chasm in the provision of patient-centered care when it comes to maternal cannabis use due to a transition to the fetus being the patient and a dissociation from the pregnant person as a patient as well. Providers saw themselves as protectors of fetal health, rather than both maternal and fetal health, with the underlying assumption that pregnant individuals were incapable of this role due to their cannabis use. This led to individuals feeling deprioritized in their provider-patient relationship. In response, pregnant patients who are protecting themselves against ambiguous and far-reaching structural consequences (e.g., CPS involvement) often will not disclose cannabis use to providers and face consequences when they do (33). Future perinatal cannabis research and educational interventions should expand the focus to encompass maternal needs as well as social and environmental experiences to keep pregnant individuals engaged.

Both patients and providers reported seeking out information on maternal cannabis use due to their lack of personal knowledge on the topic. Our findings are consistent with prior research, which shows that providers often do not adequately counsel patients following disclosure of maternal cannabis use (59). When providers do acknowledge use, it is often a punitive approach due to perceptions of unclear evidence (32, 60). While the body of research is growing and there may be fetal impacts due to use, there appears to be no benefits to surveillance measures such as drug testing (61). Further, data showing little to no fetal harm from cannabis exposure may counter provider concerns (32).

Future empirical studies must consider social and structural determinants of health when looking at neonatal outcomes from maternal cannabis use, as higher screening occurs among pregnant patients with lower socioeconomic resources.

Our research echoes previous findings that pregnant patients are using cannabis medicinally to manage pregnancy symptoms such as nausea (59). In the absence of satisfactory provider counseling, patients relied on peers for information and advice, which reiterates previous findings that individuals value information online to learn about cannabis use during pregnancy (62, 63). Patients perceived that the maternal experience of using cannabis was pushed to the margins of the general research body, with their questions and drivers of use ignored by providers. This, in turn, led to them to conduct their own personal research. As patients pursue their own research, they can frame questions around the maternal experience, highlighting ways they may not be able to in the provider's office. However, when providers lack information on the maternal experience and utilize punitive care following cannabis disclosure, it may disincentivize disclosure and pave the way for dismissal of the research or overemphasis of peer knowledge (3, 44, 64).

There is an urgent need for multi-level harm reduction-based interventions as evidence in this area is generated. This includes: (1) provider-based interventions to highlight how harm reduction can be implemented in one-on-one patient-provider appointments, education around why patients are using cannabis, and how providers can reduce structural inequities such as increased screening among low-income patients; (2) institutional interventions, such as clear guidelines for how to approach pregnant patients who disclose cannabis use, and pathways that prioritize the patient rather than punish; and (3) peer-to-peer programming with an emphasis on health literacy, media literacy, and social media literacy to enable better navigation and comprehension of cannabis content in and outside of healthcare settings (65–70).

Providers described a missed intervention point for maternal cannabis use caregiving training in their education. Providers' maternal cannabis use assumptions can be intervened upon with direct patient care during didactic and experiential learning by working with patients using cannabis to understand their lived and living experiences. This harm reduction training via an educational praxis is vital to reducing stigma, implicit bias, and moralistic judgement. Prior studies similarly show stigma towards substance use disorders and demonstrate efficacy of stigma-reducing interventions via direct exposure or didactic training (71).

Additionally, providing clear guidelines around mandatory reporting is imperative. ACOG states that, “[s]eeking obstetric–gynecologic care should not expose a woman to criminal or civil penalties for marijuana use,” which requires clear guidelines for how to handle cannabis disclosure during prenatal care (72). The current ambiguity in guidelines leaves room for provider stigma, knowledge gaps, and individual case-by-case evaluations of cannabis screening which works against structurally vulnerable pregnant patients (29). Patients must know that when they disclose their cannabis use, they have structural protection from medicolegal consequences.

Providers face difficulties as they must remain the ultimate authority in care amidst conflicting information, but including community programming could help to ease that burden. Providers typically hold a position of power in caregiving settings as they counsel patients and set their care plan including protocols for drug screening and CPS involvement. Despite this, they are not patients' only source of information and must educate within the context of social media and peer discussions (73, 74). Engaging patients and providers via structured peer-to-peer programming initiatives can educate both groups and potentially mitigate the likelihood of providers perpetrating harm by creating a negative care atmosphere or reporting to CPS resulting in loss of parental custody. Both groups need accurate, sociocultural sensitive information using a harm reduction lens to acknowledge the realities of drivers for maternal cannabis use and avoid perpetuating further damage.

### Limitations

This research has limitations. First, our findings may not be generalizable as we interviewed patients and providers who are based in California where cannabis is medically and recreationally legal. Second, maternal health stakeholders were not randomly selected and may not be representative of all providers, especially because of the limited maternal health roles they held. Third, though providers all cared for BIPOC patients, they had limited insights specific to this group as well as the role of their own identities outside of it, content we wish to explore in future projects focusing more on race and racism (75, 76). Fourth, despite patients giving informed consent to information from their medical records, some potential participants were surprised to be contacted for a cannabis study and refuted any use. Because younger, less educated, publicly insured, patients of color are more likely to be screened for substance use during prenatal care visits and face ensuing sociolegal ramifications, future studies would benefit from using self-reporting of cannabis use, despite greater identification of cannabis use via urine toxicology testing vs. self-report (30, 77). Fifth, this study may overrepresent people confident in their cannabis decision making and disclosure to providers about using during pregnancy.

Despite these limitations, this study includes a qualitative exploratory approach to understand perspectives, behaviors, motivations, and challenges on a taboo public health subject for both pregnant patients and maternal health providers. This supports ongoing research to better understand the stigma and barriers faced by those using cannabis while engaging in perinatal care. Further, our preliminary findings support future interventions that aim to inform providers and patients, protect patients from discrimination and criminalization during pregnancy, and preserve continuity of care.

## Data Availability

The original contributions presented in the study are included in the article/**Supplementary Material**, further inquiries can be directed to the corresponding author.

## Supplementary material

Study participant characteristics - pregnant patients.

Study participant characteristics - maternal healthcare providers.

Supplementary Data Sheet 1 COREQ checklist.

Supplementary Data Sheet 2 Interview guide - pregnant patients.

Supplementary Data Sheet 3 Interview guide - maternal healthcare stakeholders.

Supplementary Data Sheet 4 Codebook - people who use cannabis during pregnancy.

Supplementary Data Sheet 5 Codebook - maternal healthcare stakeholder.
