# Supplementary material for: The unseen patient: competing priorities between patients and providers when cannabis is used in pregnancy, a qualitative study
Source: Front Glob Womens Health. 2024 Apr 18;5:1355375. doi: 10.3389/fgwh.2024.1355375 (PMC11063236; doi:10.3389/fgwh.2024.1355375)
Supplement: Supplementary file 1 — Study participant characteristics - pregnant patients. [file Table1.docx]

Table 1: Study participant characteristics (pregnant patients, N = 7)

|  | Mean (SD) / Frequency (%) |
| --- | --- |
| **Participant Characteristics** |  |
| *Age* | 27.24 (3.39) |
| *Nativity*  Non-Hispanic  US-Born Hispanic  Foreign-Born Hispanic | 3 (42.86%)  3 (42.86%)  1 (14.29%) |
| *Education*  Completed grade 12 (high school)  Some college or technical school  Completed 4 years of college | 2 (28.57%)  3 (42.86%)  2 (28.57%) |
| *Income*  Don't know  Less than $15,000  $15,000 to $29,999  $30,000 to $49,999 | 2 (28.57%)  1 (14.29%)  2 (28.57%)  2 (28.57%) |
| *Preferred Language*  English | 7 (100%) |
| *Hispanic Ethnicity*  No  Yes | 3 (42.86%)  4 (57.14%) |
| *NIH Race Categories / Ethnicity*  Black, non-Hispanic  Hispanic | 3 (42.86%)  4 (57.14%) |
