# Supplementary material for: The unseen patient: competing priorities between patients and providers when cannabis is used in pregnancy, a qualitative study
Source: Front Glob Womens Health. 2024 Apr 18;5:1355375. doi: 10.3389/fgwh.2024.1355375 (PMC11063236; doi:10.3389/fgwh.2024.1355375)
Supplement: Supplementary file 2 — Study participant characteristics - maternal healthcare providers. [file Table2.docx]

Table 2: Study participant characteristics (maternal healthcare providers, N = 10)

|  | n | (n%) |
| --- | --- | --- |
| Maternal Health Provider, cares for BIPOC (Black, Indigenous, and People of Color) pregnant people | 10 | 100% |
| **Cares for** |  |  |
| People who use cannabis during pregnancy | 10 | 100% |
| People who use alcohol or other substances during pregnancy | 9 | 90% |
| People who use cannabis after pregnancy | 10 | 100% |
| Maternal Health Role: |  |  |
| Doula/Lactation Consultant | 1 | 10% |
| Certified Nurse Midwife | 1 | 10% |
| Physician | 8 | 80% |
| **Specialty** |  |  |
| OB/GYN | 9 | 90% |
| Labor and delivery | 2 | 20% |
| Postpartum | 2 | 20% |
| Community health | 1 | 10% |
| Midwifery | 1 | 10% |
| Doula | 1 | 10% |
| Lactation Consultant | 1 | 10% |
| **Maternal Health Provider's Racial Identity** |  |  |
| Black or African American | 2 | 20% |
| White | 7 | 70% |
| Mixed Race | 1 | 10% |
| **Maternal Health Provider's Pronouns** |  |  |
| He/him | 2 | 20% |
| She/her | 7 | 70% |
| Declined to disclose | 1 | 10% |
| **Maternal Health Provider's Age** |  |  |
| Under 30 | 2 | 20% |
| 30-39 | 3 | 30% |
| 40-49 | 3 | 30% |
| 50-59 | 2 | 20% |
