# Supplementary material for: The unseen patient: competing priorities between patients and providers when cannabis is used in pregnancy, a qualitative study
Source: Front Glob Womens Health. 2024 Apr 18;5:1355375. doi: 10.3389/fgwh.2024.1355375 (PMC11063236; doi:10.3389/fgwh.2024.1355375)
Supplement: Supplementary Data Sheet 1 — COREQ checklist. [file Datasheet1.docx]

Appendix 1: COREQ Checklist

| **Domain 1: Research team and reﬂexivity** |  |
| --- | --- |
| *Personal Characteristics* |  |
| 1. Interviewer/facilitator: Which author/s conducted the interview or focus group? | The leading author conducted the interviews. |
| 2. Credentials: What were the researcher’s credentials? (e.g., PhD, MD) | Authors have a BA/BS, MPH, ScD, or PhD degrees. |
| 3. Occupation: What was their occupation at the time of the study? | Authors are advanced master’s or doctoral-level students, staff members, or faculty. |
| 4. Gender: Was the researcher male or female? | There was a total of 2 interviewers; all authors are female. |
| 5. Experience and training: What experience or training did the researcher have? | Researchers were trained over a 16-week period in qualitative methods as part of a doctoral-level public health course and in fulfillment of learning qualitative methods to conduct their research projects. |
| *Relationship with participants* |  |
| 6. Relationship established: Was a relationship established prior to study commencement? | Pregnant people were part of a larger study and recruited by the study coordinator.  Researchers emailed providers based on professional contacts established with physicians and investigators, who provided introductions. Other participants were emailed for participation based on “cold emails.” |
| 7. Participant knowledge of the interviewer: What did the participants know about the researcher? (e.g. personal goals, reasons for doing the research) | Interviewer provided a brief overview of the study objectives via email and researchers provided an additional brief overview of the study objectives and purpose prior to the beginning of interviews. |
| 8. Interviewer characteristics: What characteristics were reported about the interviewer/facilitator? (e.g., bias, assumptions, reasons and interests in the research topic) | Researchers did not have any opinions on caring for patients who use of cannabis during pregnancy. |

| **Domain 2: Study design** |  |
| --- | --- |
| *Theoretical framework* |  |
| 9. Methodological orientation and Theory: What methodological orientation was stated to underpin the study? (e.g., grounded theory, discourse analysis, ethnography, phenomenology, content analysis) | Grounded theory methods and analysis were used. |
| *Participant selection* |  |
| 10. Sampling: How were participants selected? (e.g., purposive, convenience, consecutive, snowball) | We used purposeful sampling to generate an information-rich group of healthcare providers with experience in caring for people who use cannabis during pregnancy  Pregnant individuals were recruited as part of a larger study. |
| 11. Method of approach: How were participants approached? (e.g., face-to-face, telephone, mail, email) | We invited maternal health stakeholders to participate via email followed by a REDCap link, a scheduling link, and Zoom invite with link.  Pregnant individuals were approached by the larger study coordinator with an invitation to participate. |
| 12. Sample size: How many participants were in the study? | 10 maternal health stakeholders and 7 pregnant individuals. |
| 13. Non-participation: How many people refused to participate or dropped out? Reasons? | No providers refused to participate. We had one participant no show and while we followed up with them to reschedule, we did not receive a response or learn their reason for not continuing with the study.  We contacted 22 eligible patients to participate, and 7 individuals were interested. No one dropped out of the study. |
| *Setting* |  |
| 14. Setting of data collection: Where was the data collected? (e.g., home, clinic, workplace) | We collected data remotely via HIPAA-compliant Zoom. |
| 15. Presence of non-participants: Was anyone else present besides the participants and researchers? | Data was collected remotely; thus, the primary people present during the interviews were the participant and interviewers. Due to data collection remotely during the COVID-19 pandemic and often outside of clinical settings, we sometimes saw family members (e.g., spouses, young children) present in the background of Zoom meetings. |
| 16. Description of sample: What are the important characteristics of the sample? (e.g., demographic data, date) | Characteristics are detailed in Table 1 and Table 2 |
| *Data collection* |  |
| 17. Interview guide: Were questions, prompts, guides provided by the authors? Was it pilot tested? | We created a semi-structured interview guide based upon qualitative and quantitative literature, and modifications based on team feedback before and after interviews. |
| 18. Repeat interviews: Were repeat interviews carried out? If yes, how many? | We did not conduct repeat interviews as part of the study process. |
| 19. Audio/visual recording: Did the research use audio or visual recording to collect the data? | We recorded using HIPAA-compliant Zoom, which automatically produced audio and video outputs. Upon transcription of the audio file by an external transcriber, both audio and video outputs were deleted. |
| 20. Field notes: Were ﬁeld notes made during and/or after the interview or focus group? | Lead and secondary interviewers took notes during interviews, wrote brief interview summaries after interviews, and upon transcription of the interview, wrote initial memos for each interview to capture emerging themes and patterns. |
| 21. Duration: What was the duration of the interviews or focus group? | Interviews lasted between 30 and 60 minutes. |
| 22. Data saturation: Was data saturation discussed? | We discussed data saturation, and this was achieved. |
| 23. Transcripts returned: Were transcripts returned to participants for comment and/or correction? | Because we conducted interviews remotely and had an external professional transcribe the interviews, we did not return transcripts to participants for correction as we are confident in the accuracy of the transcripts. |

| **Domain 3: analysis and ﬁndings** |  |
| --- | --- |
| *Data analysis* |  |
| 24. Number of data coders: How many data coders coded the data? | Two people coded each transcript. Team members met regularly to ensure intercoder reliability and understanding of the codebook. |
| 25. Description of the coding tree: Did authors provide a description of the coding tree? | We describe the coding process in the Methods section and provide the final codebook in the Appendix. Authors developed an initial codebook used to code one transcript as a team. Based on team discussions, the codebook was iteratively revised for accuracy and consistency. |
| 26. Derivation of themes: Were themes identiﬁed in advance or derived from the data? | We identified themes derived from the data. |
| 27. Software: What software, if applicable, was used to manage the data? | ATLAS.ti, Mac Version 22.1.0 |
| 28. Participant checking: Did participants provide feedback on the ﬁndings? | No. |
| *Reporting* |  |
| 29. Quotations presented: Were participant quotations presented to illustrate the themes/ﬁndings? Was each quotation identiﬁed? (e.g., participant number) | We presented direct participation quotes in the Results section and identified participants by participant number. |
| 30. Data and ﬁndings consistent: Was there consistency between the data presented and the ﬁndings? | There was consistency between the data presented and the findings. |
| 31. Clarity of major themes: Were major themes clearly presented in the ﬁndings? | These major themes and patterns are outlined in the Results section. |
| 32. Clarity of minor themes: Is there a description of diverse cases or discussion of minor themes? | These minor themes and patterns are also outlined in the Results section and contribute to nuanced stakeholder responses. |
